# A cost-effective method for Illumina small RNA-Seq library preparation using T4 RNA ligase 1 adenylated adapters

**DOI:** 10.1186/1746-4811-8-41

**Published:** 2012-09-20

**Authors:** Yun-Ru Chen, Yi Zheng, Bao Liu, Silin Zhong, Jim Giovannoni, Zhangjun Fei

**Affiliations:** 1 Boyce Thompson Institute for Plant Research, Cornell University, Ithaca, NY, 14853, USA; 2 Northeast Normal University, Changchun, China; 3 USDA Robert W. Holley Center for Agriculture and Health, Ithaca, USA

**Keywords:** Small RNA sequencing, Directional mRNA sequencing, 3’ RNA adapter, Adenylation, T4 RNA ligase 1

## Abstract

**Background:**

Deep sequencing is a powerful tool for novel small RNA discovery. Illumina small RNA sequencing library preparation requires a pre-adenylated 3’ end adapter containing a 5’,5’-adenyl pyrophosphoryl moiety. In the absence of ATP, this adapter can be ligated to the 3’ hydroxyl group of small RNA, while RNA self-ligation and concatenation are repressed. Pre-adenylated adapters are one of the most essential and costly components required for library preparation, and few are commercially available.

**Results:**

We demonstrate that DNA oligo with 5’ phosphate and 3’ amine groups can be enzymatically adenylated by T4 RNA ligase 1 to generate customized pre-adenylated adapters. We have constructed and sequenced a small RNA library for tomato (*Solanum lycopersicum*) using the T4 RNA ligase 1 adenylated adapter.

**Conclusion:**

We provide an efficient and low-cost method for small RNA sequencing library preparation, which takes two days to complete and costs around $20 per library. This protocol has been tested in several plant species for small RNA sequencing including sweet potato, pepper, watermelon, and cowpea, and could be readily applied to any RNA samples.

## Introduction

The rapidly increasing NextGen sequencing capacity is enabling researchers to combine more samples for multiplexed sequencing, and the sequencing cost itself could be less than that of the library preparation [1]. We have previously reported a high-throughput RNA sequencing strategy that has significantly lowered the library preparation cost [2]. Here we sought to develop an alternative protocol that would also simplify the small RNA library preparation. We note that both the Illumina small RNA sequencing and directional mRNA sequencing library construction protocols are based on the early microRNA cloning strategies [3], in which two adapters are sequentially attached to the RNA molecule to create the priming sites for subsequent PCR amplification. The 5’ adapter is a conventional RNA oligo that can be synthesized by commercial oligo manufacturers. The 3’ adapter is a modified DNA oligo containing a 5’5’-adenyl pyrophosphoryl moiety and a 3’ blocking group such as amine or dideoxylnucleotides. The adenyl modification on 3’ adapter is crucial for the library preparation as it enables the adapter to be ligated to RNA in the absence of ATP by a truncated form of T4 RNA ligase 2. This effectively prevents small RNA self-ligation and concatenation. Despite the simplicity of this method, the adenylated DNA oligos are very costly to synthesize, and few are available commercially.

It has been reported that T4 DNA ligase can convert DNA oligos to pre-adenylated adapters [4]. In a ligation reaction, T4 DNA ligase first adenylates the donor DNA that has a 5’ phosphate, and the adenylated intermediate reacts with the acceptor DNA with a free 3’ hydroxyl group, resulting in phosphodiester bond formation. This process could be interrupted, allowing the adenylated intermediate to be purified. However, the adenylation efficiency of T4 DNA ligase is inconsistent and several improvements have been suggested to optimize the reaction [5]. We reason that the difficulties of utilizing T4 DNA ligase to generate adenylated adapters are partly due to the fact that it works best on double-stranded substrate, while the desired adapter is a single-stranded molecule. Hence, an RNA ligase could be a more suitable enzyme for adapter adenylation, since RNA ligation also generates a 5’ adenylated donor intermediate [6].

In this report, we demonstrate that T4 RNA ligase 1 can efficiently adenylate DNA oligos with 5’ phosphate and 3’ blocking group, and we have optimized the reaction condition for large-scale adapter production. Using the enzymatically adenylated adapters, we developed a rapid and cost-effective protocol to generate small RNA sequencing libraries.

## Materials and methods

### Equipment

Agarose gel electrophoresis system

Polyacrylamide gel electrophoresis system

Thermocycler

Qubit Fluorometer (Invitrogen)

### Reagents

T4 RNA liagse 2, truncated (NEB)

50% PEG8000 (NEB)

10 mM ATP (NEB)

RNase Inhibitor (Promega)

T4 RNA ligase 1 (Enzymatics)

SuperScriptIII Reverse Transcriptase (Invitrogen)

Phusion Hotstart DNA Polymerase (NEB)

MinElute Gel Extraction kit (Qiagen)

Quant-iT HS DNA assay kit (Inivtrogen)

### 5’ adapter RNA oligo

5’ adapter: GUUCAGAGUUCUACAGUCCGACGAUC

### 3’ adapter oligos used for adenylation

3’ non-multiplex adapter (BL1): Phosphate-CTGTAGGCACCATCAAT-amine.

3’ multiplex adapter: Phosphate-CAGATCGGAAGAGCACACGT-amine.

Substrate dA: Phosphate-ACTTCGTATGCCGTCTTCTGCTT-amine

Substrate dT: Phosphate-TAGTCGTATGCCGTCTTCTGCTT-amine.

Substrate dC: Phosphate-CTGTCGTATGCCGTCTTCTGCTT-amine.

### Reverse transcription primers

Non-multiplex RT primer: amine-ATTGATGGTGCCTACAG.

Multiplex RT primer: amine-ACGTGTGCTCTTCCGATCTG.

### Library PCR amplification primers

Universal forward PCR primer (* indicates phosphothio modification): AATGATACGGCGACCACCGAGATCTACACGACAG GTTCAGAGTTCTACAGTCCGACGAT*C.

Non-multiplex reverse PCR primer: CAAGCAGAAGACGGCATACGAGATTGATGGTGCCTACA*G.

Multiplex reverse PCR primers with 6 nt barcode (NNNNNN indicates the location of TruSeq index sequences):

CAAGCAGAAGACGGCATACGAGAT-(NNNNNN)-GTGACTGGAGTTCAGACGTGTGCTCTTCCGATC*T.

## Protocol

### Adapter adenylation

1. Dissolve the 5’ phosphorylated and 3’ blocked DNA oligo in RNase-free water to a final concentration of 100 uM.

2. Setup a reaction in 0.2 ml PCR tube containing 10 uL oligo, 10 uL 10x T4 RNA ligase 1 buffer, 40 uL 50% PEG8000, 35 uL RNase-free water and 5 uL T4 RNA ligase 1, and incubate overnight at room temperature.

3. Optional: setup multiple reactions if more adenylated adapters are required.

4. Purify the adenylated adapter using a Sephadex G-25 or silica-based spin column, e.g., Qiagen Nucleotide Removal Kit. Adjust the adapter concentration to 10 uM with RNase-free water. Note: In this reaction, T4 RNA ligase 1 will first adenylate the 5’ phosphorylated DNA oligo and attempt to join it with a suitable acceptor molecule with a 3’ hydroxyl group. The 3’ end of the oligo is block with amine and could not take part in the ligation reaction, leading to accumulation of the adenylated intermediate.

### 3’ adapter ligation

**Figure 1:**
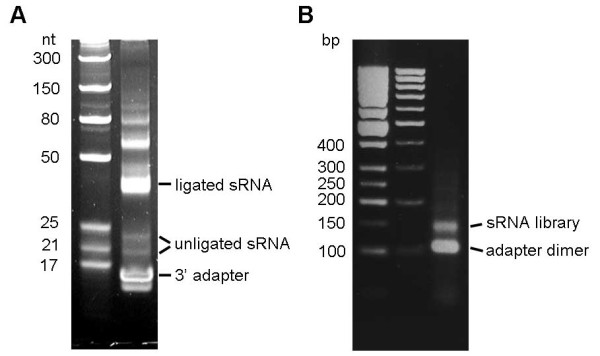
**Small RNA ligated to 3’ adapter and the PCR amplified library.** (**A**) The 3’ ligation reaction was separated on a 10% urea PAGE gel showing the adapters, small RNAs and 3’ ligation products. NEB RNA ladders are shown in the left lane. (**B**) Small RNA library after PCR amplification was separated on a 2% agarose gel. The library and adapter dimers are indicated by arrows. The 50 bp and 1 kb DNA ladders are shown on the left and middle lanes, respectively.

5. Denature the sRNA sample at 65°C for 30 sec, immediately chill on ice.

6. Denature the adenylated 3’ adapter at 65°C for 30 sec, immediately chill on ice.

7. Setup the ligation reaction with 8 uL small RNA, 1 uL adapter (10 uM), 2 uL 10x T4 RNA ligase buffer without ATP, 0.5 uL RNase inhibitor, 5 uL PEG8000 and 0.5 uL T4 RNA ligase 2, truncated. The reaction is incubated at 25°C for 4 h or 18°C overnight. Note: ligation reaction must be performed in the absence of ATP to prevent self-ligation of the small RNA that has a 5’ phosphate. Successfully ligated small RNA could be visualized on Urea-PAGE (Figure 1A).

8. Add 0.5 uL reverse transcription primer (20 uM) to the ligation reaction, heat inactivate the reaction at 65°C for 15 min and incubate the reaction at 25°C for 10 min.

9. Optional: to minimize adapter dimer formation in the subsequent PCR enrichment, the ligated sRNA could be PAGE purify before proceeding to the following 5’ adapter ligation steps.

### 5’ adapter ligation

10. Denature the 5’ adapter (10 uM, dissolved in RNase-free water) at 65°C for 30 sec, immediately chill on ice.

11. Setup the 5’ ligation reaction with 20.5 uL 3’ ligation product, 1 uL 5’ adapter (10 uM), 1 uL 10x T4 RNA ligase 1 buffer with ATP, 0.5 uL RNase Inhibitor, 2 uL 10 mM ATP, 4 uL RNase-free water and 1 uL T4 RNA ligase 1. The reaction is incubated at 25°C for 4 h or 18°C overnight. Note: the 3’ end of the small RNA has already been ligated to the 3’ adapter that has an amine group at the 3’ end, and could no longer take part in the ligation reaction; thus its 5’ end could be ligated to an RNA oligo in the presence of ATP.

### Reverse transcription

12. Add 2 uL dNTP (10 mM each), 3 uL DTT (100 mM), 9 uL 5xSuperScriptIII Frist Strand reaction buffer, and 1 uL SuperScriptIII reverse transcriptase to the 5’ ligation reaction.

13. Incubate the reaction at 50°C for 1 h, and heat inactivate the reverse transcriptase at 75°C for 15 min.

### PCR enrichment

14. Assemble the PCR reaction with 10 uL RT product, 1 uL forward primer, 1 uL reverse primer, 1 uL dNTP, 6 uL 5x Phusion HF buffer, 10.5 uL water and 0.5 uL Phusion DNA polymerase. The remaining RT product could be stored at −20°C.

15. Perform 12 to 15 cycles of PCR amplification as followed: 94°C 2 min, 12–15 cycles of 98°C for 12 sec, 65°C for 30 sec and 72°C for 30 sec, and a final extension at 72°C for 2 min.

16. Seperate the PCR product on a 2% agarose gel. Note: The multiplex sRNA-Seq library and adapter dimer are 150 and 120 bp respectively (Figure 1B); whereas the reverse transcription and PCR primers used in the Illumina non-multiplex library preparation lack the barcode sequences, hence the sizes of non-multiplex sRNA-Seq library and adapter dimer are smaller at 120 and 90 bp, respectively.

17. Cut the band corresponding to the small RNA library and gel-purify the product with Qiagen MinElute kit.

18. Optional step: adjust PCR cycles accordingly to avoid over or insufficient PCR amplification.

### Quantify and pool libraries for sequencing

19. Quantify the purified library using QuaniT HS DNA assay kit.

20. Use 10 uL of library with a final concentration no less than 2 ng/ul for each lane of Illumina sequencing.

21. Optional: if multiplex sRNA-Seq libraries were used, mix equal amount (10 ng) of each libraries, concentrate using Qiagen MinElute kit, and use 10 uL library with a final concentration no less than 2 ng/ul for each lane of Illumina sequencing.

## Comments

**Figure 2:**
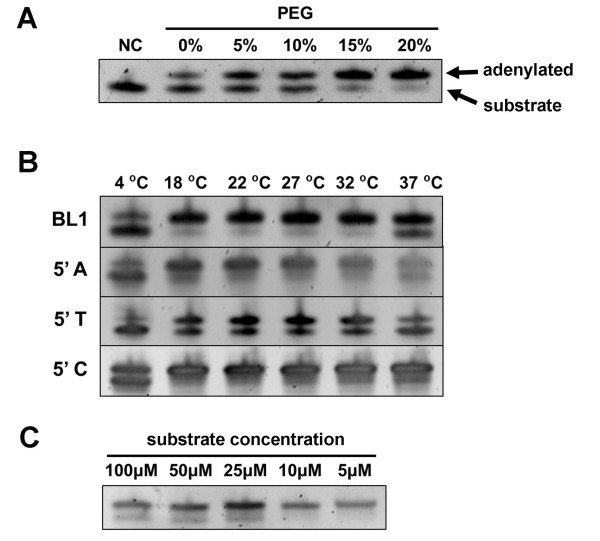
**Adenylation of 3’ adapter using T4 RNA ligase 1.** (**A**) Effect of PEG8000 concentration on adenylation efficiency. A synthetic oligo BL1 mimicking the first small RNA cloning linker reported by Lau et al. (2001) was adenylated overnight with 1 U/μL T4 RNA ligase at various PEG concentration. Non-adenylated oligo as the negative control (NC) is loaded on the left lane. (**B**) Effect of temperature and 5’ nucleotide composition on adenylation efficiency. Oligos were adenylated overnight in the presence of 20% PEG8000 at various temperatures. (**C**) Impact of oligo concentration on adenylation efficiency. Substrates with different concentrations were adenylated overnight with 20% PEG8000 at room temperature. All adenylation products were analyzed on the 20% denatured PAGE, stained with SYBR-Gold and photograph under UV.

**Figure 3:**
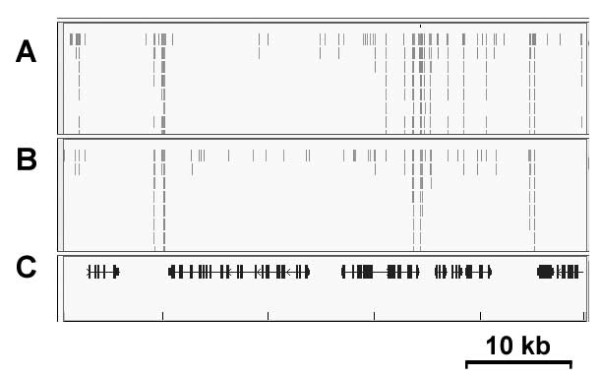
**Genome browser screenshot showing small RNA sequencing reads aligned to the tomato genome.** (**A**) Tomato fruit small RNA-Seq library prepared using the enzymatically adenylated adapter. (**B**) Published tomato small RNA-Seq data. (**C**) Genome browser track showing gene annotation. The region from 20 kb to 70 kb on tomato chromosome 1 was shown (Scale bar = 10 kb).

It has been shown that T4 RNA ligase 1 could be used to adenylated RNA oligos [7,8]. To examine its adenylation efficiency on DNA substrates, we have synthesized a 5’ phosphorylated and 3’ amine blocked single-stranded DNA substrate (p-CTGTAGGCACCATCAAT-amine) according to the first published microRNA cloning adapter [3]. Since its 3’ end is blocked by the amine modification, when its 5’ end is activated by adenylation, there is no free 3’ hydroxyl group available for ligation, leading to accumulation of the adenylated intermediate. We incubated the substrate with T4 RNA ligase 1 under various reaction conditions, and in all cases, a clear band shift was observed on the denatured PAGE indicating successful adenylation (Figure 2). We found that adenylation reactions at room temperature with 10 μM of substrate and in the presence of 20% PEG gave the best result. It has been shown that T4 RNA ligase 1 adenylates ribonucleotides with different efficiencies, and rCTP is its best substrate [6-8]. We examined its ability to act on DNA substrates with different 5’ deoxylnucleotides, and found that oligos starting with a 5’ dC were efficiently adenylated (Figure 2B). Hence we have designed all 3’ adapters with a 5’ dC to maximize the adenylation efficiency. Unlike the T4 DNA ligase-based method that requires time-consuming PAGE separation of the adenylated product from other oligos used in the reaction, oligos adenylated by T4 RNA ligase 1 can be directly purified using a Sephadex buffer exchange column or commercial kits. We have generated a sRNA-Seq library for tomato fruit using the T4 RNA ligase 1 adenylated adapter, and the result matches the published tomato small RNA datasets [9] generated by Illumina reagents (Figure 3). This protocol has also been adopted for small RNA sequencing in different plant species including sweet potato, pepper, watermelon, and cowpea.

## Competing interests

The authors declare no competing interests.

## Authors’ contributions

ZF and SZ devise the project and wrote the manuscript. YC performed the experiment and YZ, BL and JJG performed the analysis and contributed to the manuscript writing. All authors approved the final manuscript.
